# Cyclonite-Induced Seizures After Voluntary C-4 Ingestion

**DOI:** 10.7759/cureus.47746

**Published:** 2023-10-26

**Authors:** Ton Fang, Paramesh V Karandikar, Taylor R Young, Raffaella P Umeton

**Affiliations:** 1 Neurology, University of Massachusetts Memorial Medical Center, Worcester, USA; 2 Neurology, University of Massachusetts Medical School, Worcester, USA; 3 Neuropsychiatry, University of Massachusetts Memorial Medical Center, Worcester, USA

**Keywords:** ingestion, c4, military, poisoning, seizure, cyclonite

## Abstract

Cyclonite (cyclotrimethylenetrinitramine, RDX, hexogen) is the active agent in the plastic explosive, composition 4 (C-4). It has been used globally since the Vietnam War for both military and civilian applications due to its metastable nature. Ingestion or inhalation of C-4 can cause euphoric effects such as those commonly seen with alcohol toxicity, in addition to seizures and rarely fulminant liver and kidney failure. We report the case of a patient who ingested 75 g of C-4 and presented with a generalized tonic-clonic seizure four hours after ingestion. Our patient made a full recovery after being stabilized with temporizing anticonvulsants in the intensive care unit.

## Introduction

Composition 4, commonly known as C-4, is a malleable explosive material widely used in both military and civilian demolition applications due to its metastability, modifiability, and economy of production. The material is 91% cyclonite (cyclotrimethylenetrinitramine, also known as RDX, hexogen) by mass and exists in combination with plasticizers and binding agents [1].

While cyclonite saw use in weapons during the Second World War, C-4 was first fielded for widespread military use during the Vietnam War. Shortly thereafter, reports of toxicity associated with C-4 first appeared [1]. Due to its metastable nature and putty-like consistency, soldiers would frequently burn the material to heat rations in the field and risk incidental fume inhalation or food contamination [1]. It was then discovered that consuming small quantities resulted in a euphoric sensation [1]. As such, cyclonite toxicity resulted in a small but noticeable influx of patients presenting with new-onset seizures and occasionally with hepatic or renal impairment. United States military physicians reported as many as four cases of C-4 toxicity per month at a particular field hospital during the Vietnam War [2]. Such reports are extremely rare in civilian contexts. However, it is unclear how many of these presentations were attributed to accidental or voluntary exposure.

We present the case of a healthy male active-duty serviceman who presented with new-onset generalized tonic-clonic seizures after consuming 75 g of C-4 explosive.

## Case presentation

A 30-year-old healthy male in the United States military presented to the emergency department (ED) after having a generalized tonic-clonic seizure following ingestion of a C-4 plastic explosive. Reportedly, he ingested a “golf ball”-sized amount of C-4, one-eighth of a standard 1.25-lb block of C-4, equivalent to 2.75 ounces or 75 g. He later reported previous asymptomatic ingestion of smaller “marble-sized” quantities of C-4 several years ago. Approximately four hours after ingestion, he had a dissociative experience that he described as an out-of-body phenomenon, followed shortly by stomach churning and a blackout. Colleagues witnessed tonic-clonic movements that lasted less than two minutes and foaming at the mouth, followed by 90 minutes of post-ictal confusion.

He was transported by ambulance to the ED and returned to mental baseline on arrival. On initial ED investigation, he was awake and alert with no neurological deficits and only superficial abrasions to his nose. He denied any prior personal seizure history. Initial vitals showed a blood pressure of 128/86 mmHg, a temperature of 36.3 °C, a respiration rate of 20 breaths/min, and an oxygen saturation of 100% on room air. The initial complete blood count and comprehensive metabolic panel were unremarkable. The initial lactate level was mildly elevated at 3.8 mmol/L and resolved with fluid resuscitation. Phosphorus was low, 1.7 mg/dL (2.5-4.5 mg/dL) and repleted in the ED. Initial creatinine kinase was normal at 173 U/L (49-348 U/L) but was mildly elevated on repeat testing at 387 U/L. Opiates, cocaine, marijuana, amphetamines, phencyclidine, benzodiazepines, barbiturate, and propoxyphene were undetectable in serum. Urinalysis was negative, and no hematuria was noted. The electrocardiogram showed normal sinus rhythm with a borderline elevated QTc of 462 ms.

Three hours after arrival at the ED, the patient had another generalized tonic-clonic seizure and was given 2 mg of lorazepam intravenously (IV). Neurology was consulted, and neurological evaluation showed brisk right bicep, patellar, and ankle tendon reflexes with a positive extensor plantar reflex on the right side. On a visual exam, the patient was found to have a deconjugated upward gaze with a slow lateral deviation of the right eye. A CT scan of the head showed no evidence of intracranial bleeding, mass lesion, or shift of midline structures. Toxicology was consulted and advised that ingestion of C-4 may lead to seizures. Due to the substance’s terminal half-life of 48 hours, the patient was admitted to the medical intensive care unit for monitoring.

**Figure 1:**
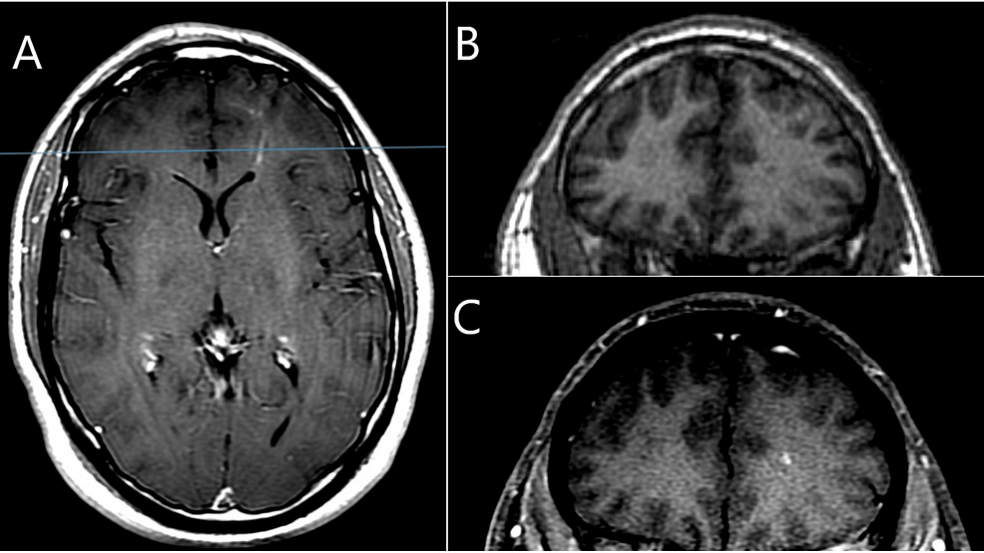
Brain magnetic resonance imaging with and without contrast identified few foci of punctate susceptibility abnormalities in the left frontal lobe (A) T1 axial sequence, contrast positive, blue line indicates area of coronal cross-section; (B) coronal SPGR sequence, contrast negative; (C) T1 coronal sequence, contrast positive.

The patient was given a small loading dose of 2000 mg of levetiracetam intravenously; subsequently, the patient was started on IV fluid overnight and given an additional 1000 mg of levetiracetam orally the following day before being tapered off with one further dose of oral 1000 mg. Neurological symptoms resolved within the first 24 hours, and no further seizure activity was observed. Brain magnetic resonance imaging with and without contrast identified foci of punctate susceptibility abnormalities in the left frontal lobe most likely related to seizure activity (Figure 1) and serpiginous contrast enhancement in the left inferior frontal lobe with a possible developmental venous anomaly.

The patient was discharged after observation and returned to military service after being cleared by a behavioral/addiction therapist. The patient had some initial discomfort after discharge, such as headaches, muscle soreness, and stomach tightness, but all symptoms resolved within one week of discharge, and he remained seizure-free without anticonvulsive medications for one year. There were no further reported symptoms.

## Discussion

Cyclonite toxicity has been reported since World War II, with variable presentations ranging from rash and gastrointestinal discomfort to generalized tonic-clonic seizures [1,3]. While hepatic and renal involvement have been reported, these tend to be self-limited, with organ failure reserved for the most severe cases [1]. Provoked seizures are reported to occur up to 60 hours after initial exposure, depending on individual bioavailability and absorption rate [1,4].

While there have been no human studies on the mechanisms of cyclonite toxicity, in vitro studies on recombinant murine tissues and in vivo studies on rodents suggest that the compound serves as a noncompetitive antagonist to GABAA receptors [5-7], which is the same receptor involved in alcohol and benzodiazepine toxicity [8]. Furthermore, binding-assay studies have demonstrated that cyclonite preferentially occupies the picrotoxin binding site of the receptor, which can lead to convulsive activity. Seizures have been provoked by the ingestion of C-4 quantities as small as 1.58 g [9], well below the amount consumed by our patient.

As such, if cyclonite toxicity is suspected, the primary goal of treatment is symptomatic management, seizure prevention, and close observation. In cases of provoked seizures, management is usually dictated by the frequency and duration of episodes, and anticonvulsant medications may be used to temporarily readjust the seizure threshold. It is unclear if our patient’s seizures would have self-resolved without the use of medications; nevertheless, his neurological exam did not return to baseline, and therefore he was treated temporarily with anticonvulsant medications. Long-term anticonvulsant prophylaxis was not indicated, as it is unlikely future seizures would occur after the removal of provoking factors, and he remained seizure-free without medication [10]. While others have found high-performance liquid chromatography assays to be useful for detecting and quantifying cyclonite in serum, this was not felt necessary in this case due to the known exposure and quality of the substance ingested [9]. Due to the varying gastrointestinal absorption and bioavailability of cyclonite, detoxification has been previously attempted with the induction of vomiting and the use of gastric binders such as charcoal, polyethylene glycol, and magnesium citrate [7], but this was decided to be of limited utility as there was a three-hour delay to the initial presentation. A further meta-analysis of similar cases may be helpful for future guidance on optimizing management strategies for patients presenting earlier during the window for gastrointestinal absorption.

While the incidence of cyclonite toxicity has diminished following the Vietnam War, reports periodically emerge of toxic ingestion of explosives by military servicemembers with readily available access to explosives. Simultaneous mass cyclonite ingestion events have also been reported during military training exercises [11,12], possibly resulting from the need for individuals to strengthen social relations, especially in a cohesive military unit [13]. Therefore, the psychological pressures amplified by the stressors and dangers of military activity could lead to voluntary ingestion during initiations, as a means of malingering, or in pursuit of euphoric effects [14-17].

Due to the inherent difficulties in proving toxic ingestion of C-4 or other substances, developing effective screening methods and detailed analysis of the causality of such events is limited, and the true incidence of subclinical voluntary cyclonite exposure is likely underreported.

## Conclusions

Cyclonite is a non-competitive antagonist of GABAA receptors. It is a rare but non-negligible cause for provoked seizures, particularly following voluntary consumption by military personnel with occupational access to C-4 plastic explosives. The use of long-term prophylactic anticonvulsants was not indicated, especially given the short half-life of the substance. High-performance liquid chromatography assays may provide early detection and quantification during C-4 ingestion.
